# Comparison of corneal tomography using a novel swept-source optical coherence tomographer and rotating Scheimpflug system in normal and keratoconus eyes: repeatability and agreement analysis

**DOI:** 10.1186/s40662-022-00290-6

**Published:** 2022-05-23

**Authors:** Robert Herber, Janine Lenk, Lutz E. Pillunat, Frederik Raiskup

**Affiliations:** Department of Ophthalmology, University Hospital Carl Gustav Carus, TU Dresden, Fetscherstraße 74, 01307 Dresden, Germany

**Keywords:** ANTERION, Cornea, Keratoconus, OCT, Pentacam, Scheimpflug, Swept-source, Topography, Tomography

## Abstract

**Background:**

To determine the repeatability and agreement using corneal tomography of a swept-source optical coherence tomographer (SS-OCT) compared to a rotating Scheimpflug camera (RSC) in normal eyes and keratoconus (KC) eyes.

**Methods:**

This prospective repeatability analysis was performed at the Department of Ophthalmology of University Hospital Carl Gustav Carus, Dresden, Germany. Forty-three normal and 57 KC eyes were enrolled in the study. Three consecutive measurements were performed by the same operator on each device. Corneal parameters of anterior and posterior corneal surface, such as simulated keratometry (SimK), as well as central and thinnest corneal thickness were evaluated. Repeatability and agreement were assessed by using the coefficient of repeatability and Bland-Altman analysis.

**Results:**

The repeatability of anterior corneal parameters was comparable between RSC and SS-OCT in normal eyes (repeatability < 0.5 D). Repeatability was increased in mild and moderate KC for all parameters using both devices. In moderate KC, repeatability of Kmax was 1.33 D and 0.78 D for RSC and SS-OCT, respectively. Repeatability of posterior corneal parameters was consistently better for SS-OCT. Significant offsets and wide ranges of limits of agreement were found between the devices for SimK and corneal thickness values.

**Conclusions:**

SS-OCT showed highly repeatable measurements of anterior and posterior corneal parameters in normal and KC eyes. Compared to RSC, the SS-OCT had a better repeatability of anterior corneal parameters in mild and moderate KC as well as posterior corneal parameters in all groups. Both devices should not be used interchangeably in the diagnostic process of patients.

*Trial registration* NCT04251143 at Clinicaltrials.gov, registered on 12 March 2018, https://clinicaltrials.gov/ct2/show/NCT04251143?cond=Keratoconus&cntry=DE&city=Dresden&draw=2&rank=1

**Supplementary Information:**

The online version contains supplementary material available at 10.1186/s40662-022-00290-6.

## Background

The assessment of corneal topography and tomography is an essential method prior to laser vision correction, intraocular lens calculation and for detection of corneal abnormalities, such as corneal ectasia. Over many years, Placido-disc videokeratoscopy was a valuable tool to measure and visualize anterior corneal curvature but with the drawback of missing information relating to posterior corneal curvature and corneal thickness [1]. A further milestone was the introduction of a rotating Scheimpflug camera (RSC) which allows tomographic visualization of the anterior segment of the eye, including the anterior and posterior shape as well as thickness of the cornea [2]. All these methods are suitable for detecting corneal abnormalities and distinguishing them from physiological variations. The early detection of corneal ectasia is critical prior to refractive surgery or to prevent loss of vision resulting from undiagnosed keratoconus or progression of any missed sub-clinical disease. Keratoconus (KC) is an ectatic disease of the cornea accompanied by stromal thinning and apical protrusion that occurs mostly in the second or third decade of life [3]. Irregular astigmatism due to ectatic changes leads to a loss of vision, which can be best corrected with rigid gas permeable lenses or scleral lenses in advanced stages [3–5]. Corneal cross-linking is the most common treatment to prevent progression of the disease [6]. The clinically relevant criteria for determining the progression of KC are the steepening of more than 1 D in maximum keratometry (Kmax) [7, 8], the increase of astigmatism by more than 1 D [8] or more than 3 D [9], and the decrease in corneal thickness by more than 5% [9] within 12 months. The Global Consensus from 2015 recommended the change in posterior curvature as an additional parameter [10]. Therefore, measurement devices must provide a fast and reliable single measurement with high precision. RSC is known to reliably measure corneal tomographic parameters; however, there is a discrepancy in repeatability of this technology in the measurement of moderate or advanced KC cases, where corneal scars may be present [11–14]. Swept-source optical coherence tomography (SS-OCT) is a novel technology to measure corneal tomography characterized by a longer wavelength (845 nm [15] or 1300 nm [16]), instead of blue light, as it is used in Scheimpflug technology (475 nm). Moreover, the SS-OCT provides a live eye tracking during the measurement and faster scanning speeds compared to RSC. The anterior segment of the eye is scanned radially with both devices modeling corneal tomography.

This study aimed to evaluate the repeatability and agreement between a novel SS-OCT compared to an established Scheimpflug tomograph in healthy and KC eyes.

## Methods

### Subjects

This study was a prospective, monocentric, observational study at the Department of Ophthalmology, University Hospital Carl Gustav Carus, Technical University, Dresden, Germany. The study was approved by the ethics committee of the Technical University Dresden following the Declaration of Helsinki. Study participants were enrolled from April 2019 to February 2022 from the refractive surgery clinic and the keratoconus clinic of the Department of Ophthalmology, University Hospital Carl Gustav Carus Dresden. All subjects had to sign the informed consent to participate in the study. They had to be aged between 18 and 45 years. To ensure a normal tomography of healthy subjects (normal eyes), they had to meet the following inclusion criteria for both eyes based on RSC measurements (Pentacam HR, software version v1.21.r51): Kmax < 47 D, inferior-superior differences in keratometry < 1.4 D, Belin/Ambrosio deviation value (BAD-D) < 1.6, KISA < 60, and a complete ophthalmologic examination without any corneal pathologies. Exclusion criteria were the inability to fixate the target light in the device, insufficient tear film or corneal reflex, any ocular diseases, previous ocular surgeries or corneal tomography outside the inclusion criteria. The inclusion criteria for KC were a Kmax > 47 D, inferior-superior differences in keratometry > 1.45 D, BAD-D > 2.6, KISA > 100, and a complete ophthalmologic examination including slit lamp biomicroscopy of the anterior segment and fundus biomicroscopy. Exclusion criteria were other corneal (e.g., pellucid marginal degeneration) and ocular diseases as well as previous ocular surgeries (e.g., cross-linking, or intrastromal ring segments). The KC group was divided into mild (BAD-D ≥ 3.0 and < 7.0) and moderate (BAD-D ≥ 7.0) subgroups according to Kreps et al. [11]. All subjects discontinued the wear of contact lenses at least 14 days before examination.

Forty-three eyes of 43 healthy subjects and 57 eyes of 57 keratoconus patients were randomly selected and examined by the same observer in a standardized order: RSC followed by SS-OCT. The randomization process was done using a specific formula in Excel software. Three consecutive measurements were performed, whereby the head was reclined from head and chin rest between each measurement. The time between the measurements amounted to 2 min. The quality criteria were sufficient if the quality score of each device evaluated the measurement as “OK” (RSC) or “pass” (SS-OCT). Measurements were repeated if a sufficient quality was not reached.

### Measurement devices

**Fig. 1:**
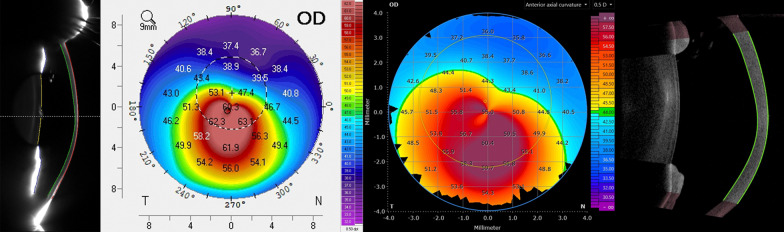
Comparison of topography maps of RSC (left) and SS-OCT (right) of a keratoconus with corresponding Scheimpflug image (left) and OCT scan (right). Color scaling is set to 0.5 D

In this study, a RSC (Pentacam HR, Oculus Optikgeräte GmbH, Wetzlar, Germany) was used to centrally illuminate the cornea with a blue slit light (475 nm) and capture 25 images from the anterior segment of the eye by Scheimpflug principle. The reference point is set to the corneal apex. The SS-OCT (ANTERION, Heidelberg Engineering GmbH, Heidelberg, Germany) uses a wavelength of 1300 nm with an in-tissue axial resolution of < 10 µm. The “Cornea App” scan mode was used to measure corneal tomography with 65 radial B-scans consisting of 256 A-scans resulting in an overall amount of 16,640 A-scans covering an 8 mm zone. The RSC uses an overall amount of 138,000 elevation points [17] based on the captured Scheimpflug images. Both RSC and SS-OCT perform a segmentation of the anterior and posterior corneal surface resulting in a 3-dimensional model of the cornea. A comparison of topography maps of the anterior corneal surface of the RSC and SS-OCT is displayed in Fig. 1. The area of central simulated keratometry (SimK) values of flat and steep meridian is 3 mm (SS-OCT) compared to 15° (RSC). From both devices, corneal curvature parameters from the anterior and posterior surface (flat SimK, steep SimK, average SimK), corneal astigmatism, Kmax and best-fit sphere (BFS) were obtained. Furthermore, central corneal thickness (CCT) and minimal corneal thickness (MCT) were analyzed as well.

### Statistical method

The data were collected using a spreadsheet software (Excel 2016, Microsoft Corp., Redmond, Washington, USA) and analyzed using R statistics (R Foundation for Statistical Computing, Vienna, Austria) as well as MedCalc (MedCalc Software Ltd, Ostend, Belgium). Repeatability was calculated based on three repeated measurements, which were performed under equal conditions without changing any factors. First, the coefficient of repeatability (repeatability) is calculated from within-subject standard deviation (Sw) based on an ANOVA model that quotes repeatability of consecutive measurements [18].

1 $${\text{repeatability}} = \surd {2 } \times { 1}.{96 } \times {\text{ Sw}} = {2}.{77 } \times {\text{ Sw}}$$

Second, the coefficient of variation (CV) is a parameter that indicates the error in percent whereas repeatability is stated in the unit of the measured variable. Thus, CV can be used to compare measuring errors between different units or devices and is calculated for each subject as follows:

2 $${\text{CV }}\left[ \% \right] = {1}00 \, \times {\text{ Sw}}/{\text{mean}}$$

Agreement between the devices was expressed with Bland-Altman plots. RSC was used as reference for the comparison with SS-OCT. MedCalc calculates the Bland-Altman plots based on the three measurements. This analysis aimed to investigate the mean differences (offset) between the measurement methods. Further, lower and upper 95% limits of agreement (95% LoA) were shown. We defined an upper and lower LoA of + / − 0.5 D (range = 1 D) and + / − 10 µm (range = 20 µm) for SimK and thickness, respectively, as clinically acceptable. Paired t tests were used to determine statistical significance of the mean differences. For statistical analysis, a *P* value lower than 0.05 was considered as statistically significant.

## Results

The demographic data of normal eyes, mild, and moderate KC are presented in Table 1.

**Table 1:** Demographics for patients with normal eyes, mild, and moderate keratoconus (KC)

Parameter	Normal eyes group	Mild KC group	Moderate KC group	*P* value
N	43	16	41	
Age (years) (mean ± SD) (min–max)	29.2 ± 7.5 (18.0–45.0)	35.6 ± 6.7 (23.0–45.0)	30.9 ± 7.4 (18.0–43.0)	0.014*
Gender (m/f) (%)	27 (63%)/16 (37%)	10 (63%)/6 (37%)	37 (90%)/4 (10%)	0.009**
Eyes (r/l) (%)	22 (51%)/21 (49%)	9 (56%)/7 (44%)	15 (37%)/26 (63%)	0.272**
Kmax (D) (mean ± SD) (min–max)	43.7 ± 1.5 (40.7–46.6)	51.5 ± 3.3 (48.1–59.7)	59.7 ± 6.8 (47.3–73.5)	< 0.001*
I-S value (D) (mean ± SD) (min–max)	− 0.01 ± 0.66 (− 1.80–1.00)	5.46 ± 1.74 (2.80–8.58)	8.66 ± 2.87 (3.23–13.92)	< 0.001*
KISA (mean ± SD) (min–max)	6.2 ± 5.9 (0.3–27.2)	440.6 ± 331.9 (101.0–1449.0)	4369.7 ± 5392.3 (104.0–25,295.0)	< 0.001*
BAD-D (mean ± SD) (min–max)	0.57 ± 0.59 ( − 0.96–1.52)	5.59 ± 0.82 (4.09–6.78)	11.23 ± 3.22 (7.14–19.86)	< 0.001*

*BAD-D* = Belin/Ambrosio deviation value; *f* = female; *I-S* = inferior-superior; *Kmax* = maximum keratometry; *KC* = keratoconus; *l* = left; *m* = male; *max* = maximum; *min* = minimum; *r* = right; *SD* = standard deviation. *One-way ANOVA; **χ^2^ test. Significant differences between groups marked in bold

### Repeatability and agreement for anterior corneal curvature parameters

The assessment of repeatability of anterior corneal curvature parameters is shown in Table 2. The repeatability for flat, steep, average SimK and astigmatism was below 0.5 D in normal eyes for RSC and SS-OCT. In the mild KC group, repeatability increased compared to normal eyes for both devices, except for BFS. The repeatability of flat SimK, steep SimK, astigmatism, and Kmax was greater than 0.5 D for RSC, whereas repeatability was lower than 0.5 D for SS-OCT. In moderate KC, an increase of repeatability of central SimK readings (flat SimK, steep SimK, and average SimK) showed a range from 0.73 to 0.92 D and from 0.48 to 0.55 D for RSC and SS-OCT, respectively.

**Table 2:** Repeatability of anterior corneal curvature parameters of all used devices

Parameter	Device	Normal eyes				Mild KC				Moderate KC			
		Mean ± SD	Sw	Repeatability	CV (%)	Mean ± SD	Sw	Repeatability	CV (%)	Mean ± SD	Sw	Repeatability	CV (%)
Flat SimK (D)	RSC	41.9 ± 1.1	0.071	0.196	0.129	43.2 ± 1.5	0.182	0.504	0.309	48.2 ± 4.8	0.283	0.784	0.414
	SS-OCT	42.1 ± 1.1	0.095	0.263	0.184	43.4 ± 1.4	0.134	0.372	0.262	47.6 ± 4.6	0.173	0.480	0.305
Steep SimK (D)	RSC	43.2 ± 1.4	0.071	0.196	0.130	46.3 ± 2	0.187	0.519	0.271	52.0 ± 4.9	0.332	0.919	0.464
	SS-OCT	43.4 ± 1.3	0.100	0.277	0.188	46.8 ± 2.0	0.145	0.402	0.271	51.9 ± 4.6	0.200	0.554	0.309
Average SimK (D)	RSC	42.5 ± 1.1	0.063	0.175	0.128	44.7 ± 1.6	0.145	0.402	0.248	50.0 ± 4.7	0.265	0.733	0.351
	SS-OCT	42.7 ± 1.1	0.089	0.248	0.163	45.0 ± 1.5	0.114	0.316	0.217	49.6 ± 4.4	0.173	0.480	0.281
Astigmatism (D)	RSC	1.2 ± 1.0	0.069	0.190	7.456	3.2 ± 1.6	0.247	0.685	5.603	3.9 ± 2.1	0.313	0.868	10.038
	SS-OCT	1.3 ± 1.0	0.082	0.229	8.618	3.4 ± 1.7	0.161	0.447	4.413	4.3 ± 2.2	0.192	0.533	4.600
Kmax (D)	RSC	43.7 ± 1.5	0.134	0.372	0.233	51.5 ± 3.3	0.224	0.620	0.369	59.7 ± 6.8	0.480	1.329	0.620
	SS-OCT	43.8 ± 1.4	0.130	0.361	0.248	50.9 ± 3.1	0.179	0.496	0.308	58.1 ± 6.1	0.283	0.784	0.377
BFS (mm)	RSC	8.0 ± 0.2	0.009	0.025	0.071	7.7 ± 0.2	0.009	0.026	0.092	7.3 ± 0.4	0.014	0.039	0.144
	SS-OCT	8.0 ± 0.2	0.010	0.028	0.099	7.6 ± 0.2	0.011	0.030	0.116	7.3 ± 0.4	0.010	0.028	0.120

*BFS* = best-fit sphere; *CV* = coefficient of variation; *Kmax* = maximum keratometry; *KC* = keratoconus; *repeatability* = coefficient of repeatability; *RSC* = rotating Scheimpflug camera; *SimK* = simulated keratometry value; *SS-OCT* = swept-source optical coherence tomography; *Sw* = within-subject standard deviation

Coefficient of repeatability of Kmax was 0.37 and 0.36 D for RSC and SS-OCT in normal eyes, respectively. Higher values of repeatability were found in mild KC for both devices, which were lower than 1.0 D. Instead, RSC showed a repeatability of 1.33 D in moderate KC that was higher than the repeatability of 0.78 D of the SS-OCT.

The increase of repeatability concerning anterior corneal parameters depended on the level of KC severity was lower using the SS-OCT in comparison to RSC.

**Fig. 2:**
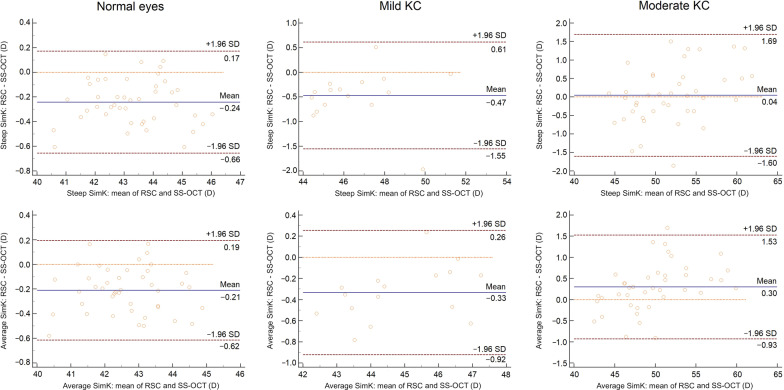
Bland-Altman plots of steep SimK (anterior), average SimK (anterior) of each subgroup

**Fig. 3:**
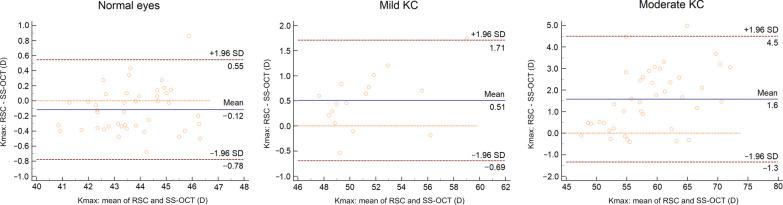
Bland-Altman plots of Kmax (anterior) of each subgroup

The mean offset between RSC and SS-OCT was mostly negative in normal and mild KC eyes with a range of 95% LoA of more than 0.7 D (Additional file 1). The offset turned into positive values indicating higher measured values by the RSC in moderate KC eyes. In addition, the offset was statistically significant for all parameters, except from astigmatism (in mild KC, *P* = 0.125) and steep SimK (in moderate KC, *P* = 0.716). In detail, the Bland-Altman plots of average SimK are given in Fig. 2. The offset of average SimK was − 0.21 D and − 0.33 D with a range of 95% LoA of 0.81 D and 1.18 D, respectively. Therefore, SS-OCT measured SimK values higher than RSC. In contrast, Kmax showed a negative offset in normal eyes, whereas a positive offset was found in mild (0.51 D, 95% LoA: − 0.69–1.71) and moderate (1.6 D, 95% LoA: − 1.34–4.5) KC eyes (Fig. 3). Further, the offset of average SimK was positive in moderate KC as well.

### Repeatability and agreement for posterior corneal curvature parameters

For the posterior corneal surface, the repeatability was below 0.5 D for all analyzed parameters for RSC and SS-OCT in all three subgroups (Table 3). Higher values of repeatability were found depending on the severity of KC using both devices. However, repeatability of the SS-OCT device was lower in all subgroups compared to the RSC.

**Table 3:** Repeatability of posterior corneal curvature parameters of all used devices

Parameter	Device	Normal eyes				Mild KC				Moderate KC			
		Mean ± SD	Sw	Repeatability	CV (%)	Mean ± SD	Sw	Repeatability	CV (%)	Mean ± SD	Sw	Repeatability	CV (%)
Flat SimK (D)	RSC	− 5.9 ± 0.3	0.035	0.098	− 0.362	− 6.2 ± 0.4	0.105	0.291	− 0.976	− 7.2 ± 0.9	0.091	0.251	− 0.926
	SS-OCT	− 5.9 ± 0.2	0.005	0.015	− 0.078	− 6.1 ± 0.4	0.017	0.048	− 0.236	− 7.3 ± 1.0	0.041	0.114	− 0.408
Steep SimK (D)	RSC	− 6.3 ± 0.2	0.028	0.077	− 0.211	− 6.8 ± 0.4	0.068	0.188	− 0.722	− 8.0 ± 1.0	0.074	0.206	− 0.734
	SS-OCT	− 6.2 ± 0.2	0.008	0.021	− 0.100	− 6.9 ± 0.5	0.020	0.055	− 0.258	− 8.1 ± 1.0	0.033	0.092	− 0.312
Average SimK (D)	RSC	− 6.1 ± 0.2	0.028	0.077	− 0.220	− 6.5 ± 0.4	0.065	0.180	− 0.726	− 7.6 ± 0.9	0.064	0.177	− 0.681
	SS-OCT	− 6.0 ± 0.2	0.005	0.015	− 0.073	− 6.5 ± 0.4	0.014	0.039	− 0.190	− 7.7 ± 1.0	0.033	0.092	− 0.340
Astigmatism (D)	RSC	− 0.4 ± 0.2	0.036	0.101	− 7.107	− 0.6 ± 0.3	0.120	0.333	− 23.142	− 0.8 ± 0.5	0.099	0.276	− 14.586
	SS-OCT	− 0.4 ± 0.2	0.009	0.025	− 2.720	− 0.8 ± 0.4	0.022	0.062	− 2.281	− 0.8 ± 0.4	0.039	0.107	− 4.616
Kmax (D)	RSC	− 6.5 ± 0.3	0.034	0.095	− 0.460	− 8.2 ± 0.7	0.160	0.443	− 1.570	− 9.9 ± 1.5	0.176	0.488	− 1.459
	SS-OCT	− 6.3 ± 0.3	0.014	0.039	− 0.188	− 8.1 ± 0.8	0.045	0.124	− 0.407	− 9.8 ± 1.5	0.071	0.196	− 0.519
BFS (mm)	RSC	6.6 ± 0.2	0.013	0.036	0.150	6.4 ± 0.2	0.016	0.045	0.213	6.1 ± 0.4	0.020	0.055	0.237
	SS-OCT	6.7 ± 0.2	0.010	0.029	0.100	6.5 ± 0.2	0.008	0.023	0.099	6.1 ± 0.4	0.010	0.028	0.161

*BFS* = best-fit sphere; *CV* = coefficient of variation; *Kmax* = maximum keratometry; *KC* = keratoconus; *repeatability* = coefficient of repeatability; *RSC* = rotating Scheimpflug camera; *SimK* = simulated keratometry value; *SS-OCT* = swept-source optical coherence tomography; *Sw* = within-subject standard deviation

Agreement of posterior corneal parameters was lower than 0.25 D and 0.15 mm for SimK values and BFS, respectively (Additional file 1). However, statistical significance was found for all parameters in normal eyes and for average SimK, astigmatism, and BFS in mild as well as moderate KC eyes (*P* < 0.05). The width of LoA was narrower than in anterior corneal parameters. However, the width of LoA increased with a higher severity of KC.

### Repeatability and agreement for corneal thickness

For RSC, the repeatability revealed a value of 4 and 4.8 µm for CCT and MCT in normal eyes, respectively (Table 4). A higher repeatability between 6.5 and 8.4 µm was found in the mild and moderate KC group for CCT and MCT, respectively. In contrast, the repeatability of SS-OCT was between 2.1 and 2.5 µm for CCT and MCT in normal as well as mild KC eyes, respectively. In the moderate KC group, the repeatability of CCT and MCT increased to 4.8 and 3.4 µm, respectively.

**Table 4:** Repeatability of corneal thickness of all used devices

Parameter	Device	Normal eyes				Mild KC				Moderate KC			
		Mean ± SD	Sw	Repeatability	CV (%)	Mean ± SD	Sw	Repeatability	CV (%)	Mean ± SD	Sw	Repeatability	CV (%)
CCT (µm)	RSC	556 ± 25	1.449	4.017	0.223	515 ± 31	2.366	6.559	0.307	466 ± 32	3.033	8.407	0.561
	SS-OCT	549 ± 26	0.775	2.147	0.097	513 ± 30	0.775	2.147	0.134	462 ± 34	1.732	4.801	0.271
MCT (µm)	RSC	553 ± 25	1.761	4.880	0.272	501 ± 30	2.387	6.618	0.402	451 ± 31	3.033	8.407	0.566
	SS-OCT	546 ± 27	0.775	2.147	0.095	493 ± 27	0.894	2.479	0.125	444 ± 30	1.225	3.395	0.196

*CCT* = central corneal thickness; *CV* = coefficient of variation; *KC* = keratoconus; *MCT* = minimal corneal thickness; *repeatability* = coefficient of repeatability; *RSC* = rotating Scheimpflug camera; *SS-OCT* = swept-source optical coherence tomography; *Sw* = within-subject standard deviation

Comparing RSC with SS-OCT, an overall positive mean offset was found for CCT and MCT in the normal, mild KC and moderate KC eyes (Additional file 1, Fig. 4). The offset ranged between 2 and 7.2 µm and showed statistical significance in normal and moderate KC eyes (*P* < 0.05). Moreover, the width of 95% LoA ranged from 23 to 29 µm as well as from 26.8 to 60.6 µm for CCT as well as MCT, respectively (Additional file 1, Fig. 4).

**Fig. 4:**
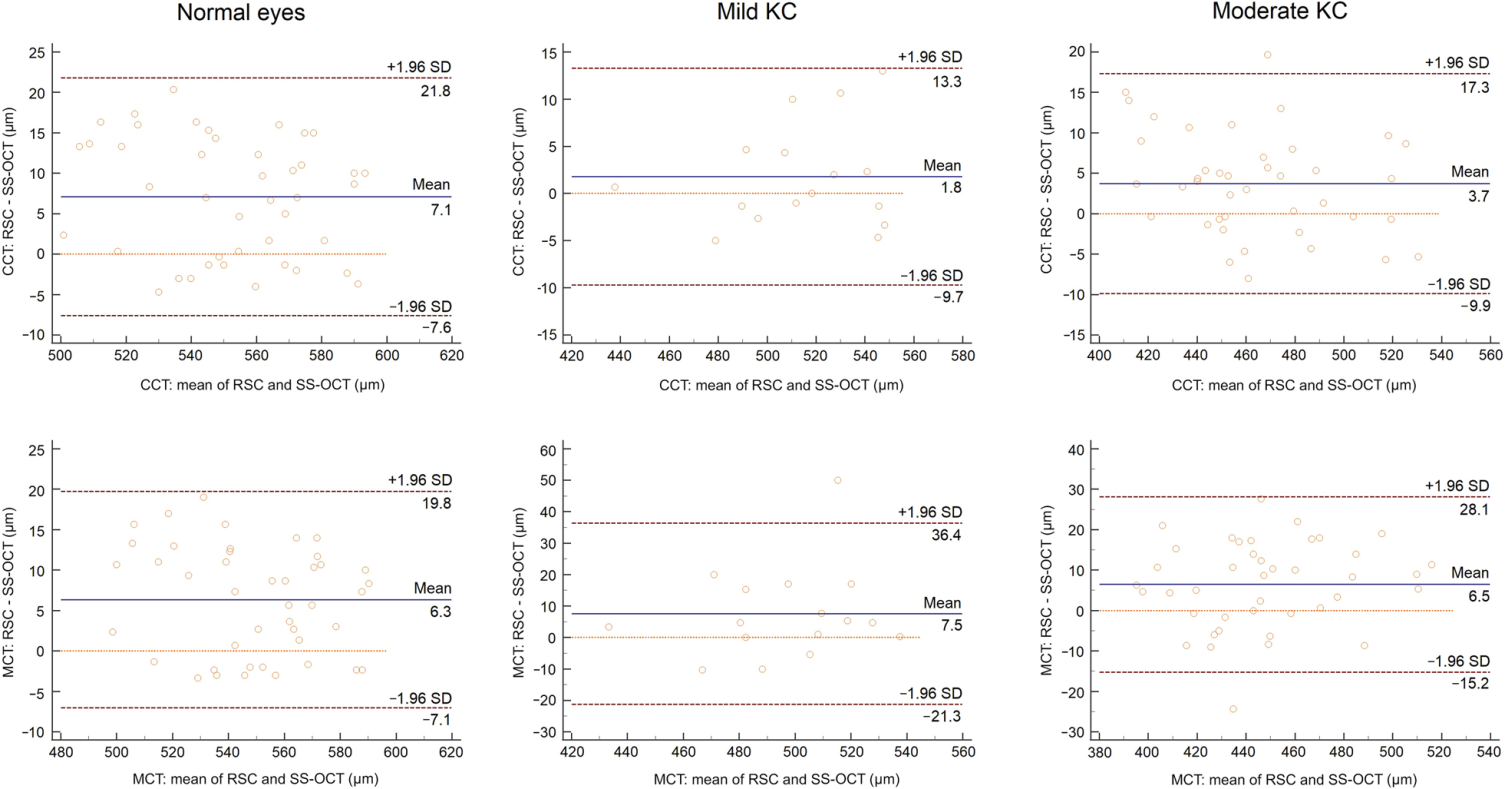
Bland-Altman plots of central corneal thickness (CCT), and minimal corneal thickness (MCT) of each subgroup

## Discussion

Repeatability and agreement analysis are fundamental investigative tools to assess the performance within and between measurement devices. Novel technologies are usually compared with the gold standard. The RSC technology is an established method to visualize the anterior segment of the eye. The RSC (Pentacam HR) has an extensive software package that allows numerous specialized analyses for keratoconus detection [19, 20] and progression [21]. The SS-OCT (ANTERION) on the other hand, is a novel device for anterior segment analysis, including a specific mode called “Cornea App” for corneal tomography measurements. This study aimed to compare both devices concerning repeatability and agreement of various corneal tomography parameters in normal and KC eyes.

The main finding of the current study revealed that the repeatability of anterior corneal parameters was comparable between RSC and SS-OCT in normal eyes with a slightly better tendency towards the RSC. In contrast, the repeatability was better using the SS-OCT in mild and moderate KC eyes. Further, the repeatability of most posterior corneal parameters was better with SS-OCT than with RSC in all subgroups. The offsets between both devices were mostly statistically significant and differed between the subgroups, ruling out interchangeability between the devices. The mean difference for Kmax was especially high in moderate KC eyes. Furthermore, wide LoA were also observed. To the best of our knowledge, this study is the first to compare RSC (Pentacam) and the novel SS-OCT (ANTERION) in normal and KC eyes with regards to the repeatability and agreement of corneal tomography parameters.

### Anterior corneal curvature parameters in normal and KC eyes

In this study, we found that higher keratoconic disorders affect repeatability of both devices negatively. In comparison to normal eyes, the repeatability of flat SimK and steep SimK was elevated in KC eyes for both devices. A similar finding was observed by Kreps et al. for RSC, in which repeatability was 0.16 D (flat SimK) and 0.2 D (steep SimK) in normal eyes and 0.63 to 0.87 D (flat SimK) as well as 0.56 to 0.82 D (steep SimK) in mild and moderate KC eyes, respectively. Here, similar criteria for subgroups were used. The repeatability of RSC was comparable for flat SimK (0.2 D) and steep SimK (0.2 D) in normal eyes. We also found elevated values for repeatability for mild (0.5 and 0.8 D) and moderate (0.5 and 0.8 D) KC eyes for flat SimK and steep SimK, respectively, which were like those reported by Kreps et al. [11].

Tañá-Rivero et al. investigated the repeatability of SS-OCT (ANTERION) in normal eyes and found the repeatability lower than 0.25 D for flat SimK, steep SimK and average SimK [22]. In this study, repeatability was found to be comparable (between 0.25 and 0.28 D). The repeatability of astigmatism was 0.18 D [22], which was lower than our findings (r = 0.23 D).

For Kmax, the repeatability was comparable for both devices in normal eyes. The repeatability of Kmax was 0.37 D, which was lower than reported values of Kosekahya et al. (r = 0.55 D) in normal eyes using the RSC [23]. Further studies which have used the RSC also found higher values for repeatability (0.47 and 0.8 D, respectively) [11, 17].

In the management of KC, the assessment of the maximum corneal curvature (Kmax) of the anterior corneal surface is commonly used to determine the disease’s progression. A change in Kmax of 1 D within 6 or 12 months is reported as a clinically significant progression [7, 8]. Therefore, the repeatability of Kmax should be lower than the defined border of clinical progression. In our study, repeatability increased for Kmax for both devices, however, the magnitude was much higher in RSC than in SS-OCT. An increase of repeatability indicates a poorer reliability. In mild KC eyes, the repeatability of RSC and SS-OCT were 0.62 and 0.5 D, respectively. In moderate KC eyes, the repeatability of RSC was 1.3 D compared to 0.8 D measured by SS-OCT indicating a lower reliability of the RSC in these higher stages of KC. Previously, several studies reported that as the repeatability increases, the higher the stage of KC is, when using RSC [11, 14, 23, 24]. In these studies, the values for repeatability ranged from 0.51 to 0.81 D, from 1.04 to 1.19 D, and from 1.34 to 1.66 D for early, moderate, and advanced KC, respectively [11, 14, 23]. The overall repeatability was reported between 0.99 [23] and 1.12 D [14]. Some of these studies concluded that the repeatability of Kmax in different stages of KC should be considered in clinical practice [11, 14]. In contrast, the repeatability of Kmax was found to be less than 1 D in KC eyes using SS-OCT in our study, leading us to conclude that a change of 1 D in Kmax as measured by SS-OCT can be used as an indicator of KC progression, but not in RSC. The steep SimK is another parameter that is considered to describe the disease’s progression [25–27]. Here, steep SimK showed repeatability lower than 1 D for mild and moderate KC for both devices. Moreover, there was no statistically significant offset observed in moderate KC leading to the assumption that this parameter could be useful for defining progression, even between different devices.

The Casia 2 (Tomey Corp., Nagoya, Japan) is another commercially available SS-OCT assessing corneal tomography. In a previous study, it was found that the standard deviation of five consecutive measurements increased with the severity of KC using both RSC and SS-OCT (Casia 2), while the outcome was in favor of RSC, except for very advanced stages of KC [24]. In contrast, our results revealed that repeatability of the anterior corneal parameters were in favor of the SS-OCT (ANTERION) in both KC subgroups with highest differences for Kmax.

The Bland-Altman analysis revealed that a (significant) negative offset was found for SimK values indicating higher SimK readings by SS-OCT in normal and mild KC eyes, except from Kmax. In contrast, SimK readings were lower for SS-OCT in moderate KC eyes than for RSC (positive offset). The LoA were extended the higher the stage of KC (greater than 1 D). The statistically significant offset does not allow interchangeability of anterior parameters in normal as well as in KC eyes.

### Posterior corneal curvature parameters in normal and KC eyes

The assessment of the posterior corneal surface became a major role in diagnosing KC and monitoring progression since it is measurable using Scheimpflug tomography [21, 28]. As mentioned, the posterior corneal curvature was considered an additional parameter for evaluating the progression of KC [10, 29]. Tellouck et al. and Fujimoto et al. showed that posterior steepening occurred earlier than anterior steepening, indicating an earlier detection of progression [30, 31]. Therefore, precise measurements of posterior corneal parameters are necessary for determining an ectatic progression.

Here, posterior corneal surface parameters demonstrated better repeatability when measured by SS-OCT than by RSC in all subgroups. The repeatability of RSC was consistently higher for flat SimK, steep SimK, average SimK, astigmatism, and Kmax in comparison to SS-OCT in normal and KC eyes. Szalai et al. found an increase of repeatability for flat SimK (0.16 vs. 0.55 D), steep SimK (0.18 vs. 0.51 D) and average SimK (0.18 vs. 0.55 D) between normal and KC eyes using RSC [32]. Furthermore, Kreps et al. reported repeatability values for central posterior SimK readings of 0.1 D in normal eyes and between 0.13 and 0.35 D in mild and moderate KC [11]. These observations were comparable to our study results, whereas repeatability values were lower compared to Szalai et al. For SS-OCT, Tañá-Rivero et al. observed similar values to those found in our study. Moreover, Flockerzi et al. found that posterior corneal parameters showed better repeatability in both healthy and KC eyes using the Casia 2 compared to the RSC [24].

The repeatability of BFS did not differ between the devices with respect to normal eyes, whereas lower values were found for SS-OCT in mild and moderate KC. The results were in tandem with previous investigations [11, 14, 22].

The Bland-Altman analysis revealed a non-significant offset as well as a range of LoA close to 1 D between both devices in mild and moderate KC eyes for posterior flat SimK, steep SimK, and Kmax, indicating good agreement. However, the range of LoA of Kmax was 1.66 and 2.27 D for mild and moderate KC, respectively, which cannot be assumed as interchangeable.

### Corneal thickness measurements in normal and KC eyes

A high repeatability was found for CCT and MCT in normal and KC eyes for both devices. The maximum of repeatability was 8.4 and 4.8 µm for RSC and SS-OCT, respectively. The CV of the measurements was < 1%. Three studies considered a corneal thickness reduction as a factor for the disease’s progression if the loss of MCT was more than 20 µm [33], 10 µm [34], or more than 5% [25]. These limits seem to be appropriate, as the measurement variability was found to be lower than 10 µm and 1% for both devices in the current study. Previously, several studies reported higher values of repeatability in normal eyes [32, 35] and KC eyes [11, 14, 36, 37], where the 10 µm criteria would not be met. Overall, the SS-OCT showed lower values of repeatability indicating higher reliability. Similar results were found comparing the SS-OCT (Casia 2) with the RSC [24]. Therefore, it is assumed that SS-OCT might be more appropriate for assessing corneal thinning in the progression period of KC. An offset between 1.7 and 7.5 µm was observed in this study between RSC and SS-OCT, where RSC measured a higher corneal thickness consistently, which was statistically significant in normal and moderate KC eyes. In addition, the wide range of more than 20 µm of LoA excludes interchangeability between the devices. Moreover, there is currently no consensus, if different technologies, such as OCT, Scheimpflug or ultrasound, when assessing corneal thickness, are interchangeable or if conversion factors are applicable. Several reports demonstrated a higher measured corneal thickness by RSC compared to OCT devices [24, 38, 39], whereas interchangeability is reported as well [40]. These discrepancies might be due to the different investigated populations and dependent on age and the presence of ocular pathologies [41]. All in all, assessing corneal thickness should be performed using the same device to evaluate changes accurately in patients.

Scheimpflug (RSC) technology was also compared with SS-OCT in corneas with specific properties following INTACS implantation as well as Fuchs endothelial corneal dystrophy (FECD). Matar et al. reported after INTACS implantation, in KC eyes, there was a higher repeatability of Kmax using the RSC (> 1 D) than using the Casia 2 SS-OCT (< 1 D) [42]. Furthermore, both the SimK and corneal thickness were found to be higher with RSC than SS-OCT, which we attributed to the different measuring methods and the insufficient detection of corneal curvature due to higher reflectivity of the implant [42]. We concluded that both devices are eligible to assess corneal tomography for long-term follow-ups, however, interchangeability is not advised [42]. Comparing the results of our study, SimK values and corneal thickness were found to be higher in KC eyes, especially in moderate KC, measured by RSC compared to SS-OCT. The findings of the repeatability of Kmax were equivalent to the findings of Matar et al. comparing both technologies. Moreover, a steeper anterior corneal curvature, a flatter posterior curvature, and a thinner corneal thickness were determined by SS-OCT (ANTERION) in comparison to RSC in FECD eyes [43].

According to the technical aspects, the better repeatability of the SS-OCT compared to the RSC might be not explained by the available scans or data points (16,640 scans vs. 138,000 elevation points). Instead, it can be assumed that the number of radial B-scans (65 vs. 25 images of the RSC), the shorter scanning time (< 1 s vs. 1 s of the RSC), and the live eye tracking system result in a more precise measurement of the SS-OCT.

Based on the results of the current study, the SS-OCT seems to be a better tool for assessing corneal tomography (SimK and corneal thickness) in follow-up examinations in normal eyes as well as mild and moderate KC eyes. Currently, the SS-OCT (ANTERION) is limited in providing a specific ectasia screening tool. As mentioned before, RSC (Pentacam) provides a wide range of screening tools, especially for corneal ectasia, keratoconus detection as well as progression. For these applications, the RSC is very helpful in clinical practice. Further, the repeatability is adequate for measuring normal and mild KC eyes.

The study is limited by a non-randomized order of the performed measurements. RSC was always measured before SS-OCT indicating that a certain learning effect could not be avoided, vice versa, patients could also become more tired after these many repeated measurements. Moreover, the age was not matched properly between all subgroups along with the normal eyes and mild KC group involving more female cases (37%) than the moderate KC group (10%). This might influence the results.

## Conclusion

The novel SS-OCT showed highly repeatable measurements of anterior, posterior, and corneal thickness parameters in normal and KC. The repeatability did not differ between SS-OCT and RSC for the majority of anterior corneal parameters in normal eyes, whereas better repeatability was achieved by SS-OCT in mild and moderate KC. Particularly, the most clinically relevant parameter Kmax showed better repeatability measured by SS-OCT than by RSC and a repeatability of less than 1 D was found for SS-OCT in mild and moderate KC. Posterior corneal parameters showed a better repeatability using the SS-OCT in all subgroups. Due to significant offsets and wide ranges of LoA between two devices, interchangeability is not recommended.

## Supplementary Information

**Additional file 1:** Bland-Altman analysis between RSC and SS-OCT of anterior and posterior corneal curvature/power and corneal thickness.

## Data Availability

The dataset used and/or analyzed during the current study are available from the corresponding author on reasonable request.
