# Supplementary material for: Comparison of corneal tomography using a novel swept-source optical coherence tomographer and rotating Scheimpflug system in normal and keratoconus eyes: repeatability and agreement analysis
Source: Eye Vis (Lond). 2022 May 23;9:19. doi: 10.1186/s40662-022-00290-6 (PMC9128256; doi:10.1186/s40662-022-00290-6)
Supplement: Supplementary file 1 — Additional file 1: Bland-Altman analysis between RSC and SS-OCT of anterior and posterior corneal curvature/power and corneal thickness. [file 40662_2022_290_MOESM1_ESM.docx]

Additional 1 Bland-Altman analysis between RSC and SS-OCT of anterior and posterior corneal curvature/power and corneal thickness

| **Parameter** | **Normal eyes** | ***P* value** | **Mild KC** | ***P* value** | **Moderate KC** | **P value** |
| --- | --- | --- | --- | --- | --- | --- |
|  | Offset (lower LoA – upper LoA) |  | Offset (lower LoA – upper LoA) |  | Offset (lower LoA – upper LoA) |  |
| Anterior | | | | | | |
| Flat SimK (D) | −0.19 (−0.64 – 0.26) | **<0.001** | −0.21 (−0.90 – 0.48) | **0.014** | 0.52 (−0.98 – 2.01) | **<0.001** |
| Steep SimK (D] | −0.24 (−0.66 – 0.17) | **<0.001** | −0.47 (−1.55 – 0.61) | **0.002** | 0.04 (−1.60 – 1.69) | 0.716 |
| Average SimK (D] | −0.21 (−0.62 – 0.19) | **<0.001** | −0.33 (−0.92 – 0.26) | **<0.001** | 0.30 (−0.93 – 1.53) | **0.002** |
| Astigmatism (D) | −0.07 (−0.42 – 0.29) | **0.009** | −0.26 (−1.59 – 1.07) | 0.125 | −0.47 (−2.41 – 1.47) | **0.003** |
| Kmax (D) | −0.12 (−0.78 – 0.55) | **0.015** | 0.51 (−0.69 – 1.71) | **0.003** | 1.58 (−1.34 – 4.50) | **<0.001** |
| BFS (mm) | 0.04 (−0.03 – 0.12) | **<0.001** | 0.06 (−0.03 – 0.16) | **<0.001** | 0.07 (−0.02 – 0.16) | **<0.001** |
| Posterior | | | | | | |
| Flat SimK (D) | −0.07 (−0.15 – 0.01) | **<0.001** | −0.07 (−0.38 – 0.25) | 0.065 | 0.04 (−0.49 – 0.57) | 0.324 |
| Steep SimK (D) | −0.09 (−0.18 – −0.01) | **<0.001** | 0.11 (−0.45 – 0.67) | 0.125 | 0.06 (−0.50 – 0.61) | 0.208 |
| Average SimK (D) | −0.08 (−0.17 – 0) | **<0.001** | 0.02 (−0.17 – 0.2) | 0.382 | 0.04 (−0.24 – 0.33) | **0.038** |
| Astigmatism (D) | −0.03 (−0.11 – 0.05) | **<0.001** | 0.17 (−0.61 – 0.96) | **<0.001** | 0.01 (−0.94 – 0.97) | **<0.001** |
| Kmax (D) | −0.14 (−0.26 – −0.02) | **<0.001** | 0.001 (−0.83 – 0.83) | 0.995 | −0.10 (−1.23 – 1.04) | 0.283 |
| BFS (mm) | −0.15 (−0.22 – −0.08) | **<0.001** | −0.10 (−0.22 – 0.03) | **<0.001** | −0.05 (−0.20 – 0.11) | **<0.001** |
| CCT (µm) | 7.09 (−7.62 – 21.79) | **<0.001** | 1.77 (−9.71 – 13.26) | 0.217 | 3.73 (−9.87 – 17.33) | **<0.001** |
| MCT (µm) | 6.35 (−7.05 – 19.75) | **<0.001** | 7.54 (−21.29 – 36.37) | 0.056 | 6.46 (−15.21 – 28.14) | **<0.001** |

BFS= best-fit sphere; CCT= central corneal thickness; LoA= limits of agreement; RSC= rotating Scheimpflug camera; SimK= simulated keratometry value; Kmax= maximum keratometry; KC= keratoconus; MCT= minimal corneal thickness; SS-OCT= swept-source optical coherence tomography. Significant differences between groups marked in bold
